# Supplementary material for: A SARS-CoV-2 variant elicits an antibody response with a shifted immunodominance hierarchy
Source: bioRxiv. 2021 Oct 13:2021.10.12.464114. Preprint. [Version 1] doi: 10.1101/2021.10.12.464114 (PMC8528074; doi:10.1101/2021.10.12.464114)
Supplement: 1 [file NIHPP2021.10.12.464114v1-supplement-1.pdf]

bioRxiv preprint doi: https://doi.org/10.1101/2021.10.12.464114; this version posted October 13, 2021. The copyright holder for this preprint (which was not certified by peer review) is the author/funder, who has granted bioRxiv a license to display the preprint in perpetuity. It is made available under aCC-BY 4.0 International license.

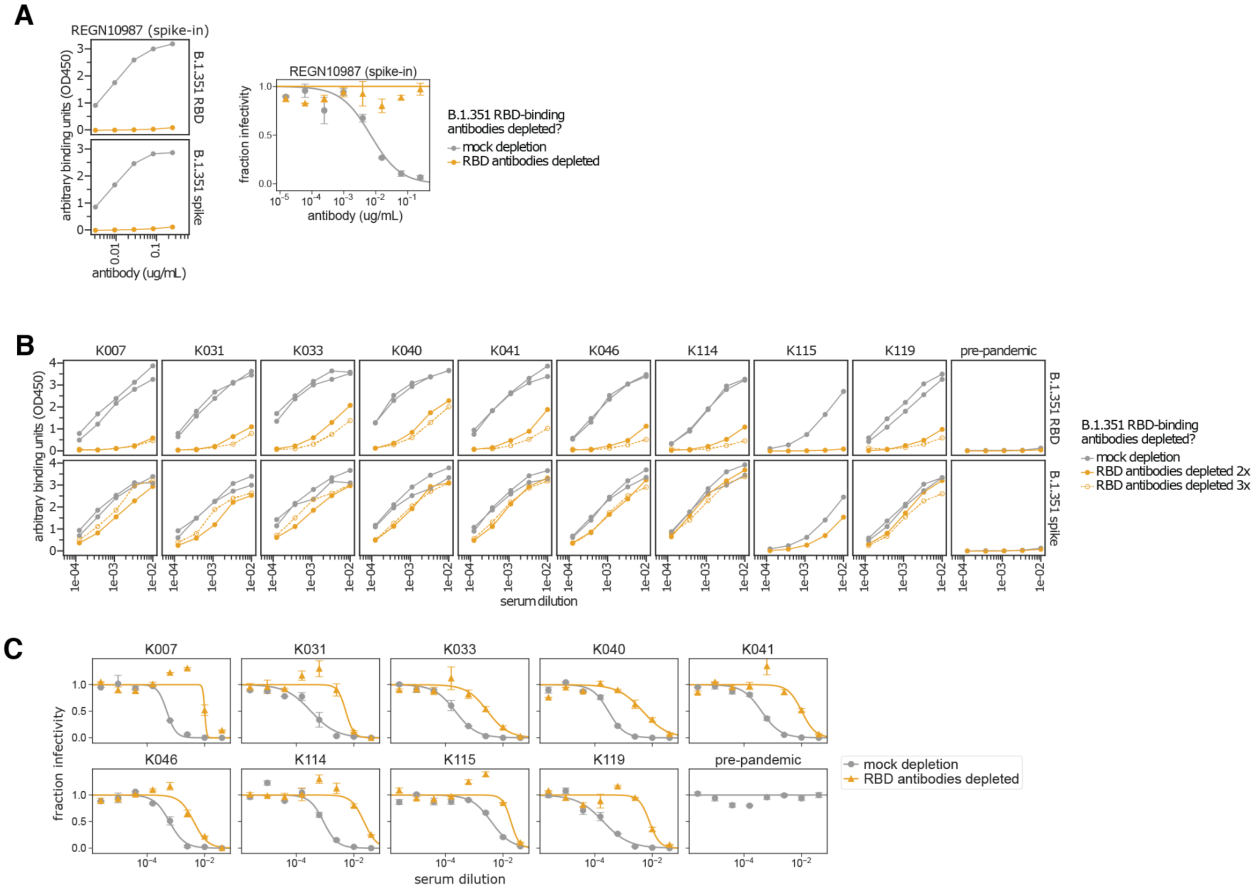

Fig. S1. Enzyme-linked immunosorbent assay (ELISA) and neutralization curves of B.1.351 convalescent plasmas before and after depletion of B.1.351 RBD-binding antibodies.
(A) Controls showing that the RBD antibody depletion completely removes a RBD-targeting neutralizing antibody. Effect of two rounds of RBD antibody depletion on binding to B.1.351 RBD and spike (left) and neutralization of B.1.351 spike-pseudotyped lentiviral particles (right) by synthetic serum. The synthetic serum was made by adding the RBD-targeting antibody REGN10987 that binds both Wuhan-Hu-1 and B.1.351 RBD (*72*) to pre-pandemic pooled serum at 50 μg/mL. The x-axis indicates the antibody concentration (μg/mL), and the y-axis is the optical density at wavelength 450 (OD450) reading at each dilution (left) or fraction infectivity (right). **(B)** Binding of B.1.351 convalescent plasmas to B.1.351 RBD and spike for mock depletion (gray lines) and depletion of RBD-binding antibodies (orange lines). Some samples were depleted three times (dashed lines and open circles) if two rounds of depletion did not abrogate binding to RBD. There were not substantial reductions in OD450 after the third round of depletions, so we reasoned that the samples were maximally depleted, and no further rounds of depletions were performed. Removal of RBD-binding antibodies only modestly reduces spike binding, consistent with prior findings that the majority of anti-spike antibodies do not bind the

bioRxiv preprint doi: https://doi.org/10.1101/2021.10.12.464114; this version posted October 13, 2021. The copyright holder for this preprint (which was not certified by peer review) is the author/funder, who has granted bioRxiv a license to display the preprint in perpetuity. It is made available under aCC-BY 4.0 International license.

RBD (*28*, *77*–*80*). **(C)** Neutralization curves for plasma mock depletion (gray circles) and depletion of RBD-binding antibodies (orange triangles). Each assay was performed in technical duplicate, and points show the mean and standard error of the replicates. Pre-pandemic pooled serum was included in (B) and (C) as a negative control for binding and neutralization. RBD-binding antibodies were removed from the plasma using streptavidin magnetic beads conjugated to biotinylated B.1.351 RBD. All binding assays were performed with B.1.351 RBD and spike, and all neutralization assays were performed with B.1.351 spike-pseudotyped lentiviral particles. This figure shows the underlying measurements for all of the B.1.351 plasmas in **Fig. 2**; the underlying measurements for the early 2020 plasmas in **Fig. 2** are shown in (*28*).

bioRxiv preprint doi: https://doi.org/10.1101/2021.10.12.464114; this version posted October 13, 2021. The copyright holder for this preprint (which was not certified by peer review) is the author/funder, who has granted bioRxiv a license to display the preprint in perpetuity. It is made available under aCC-BY 4.0 International license.

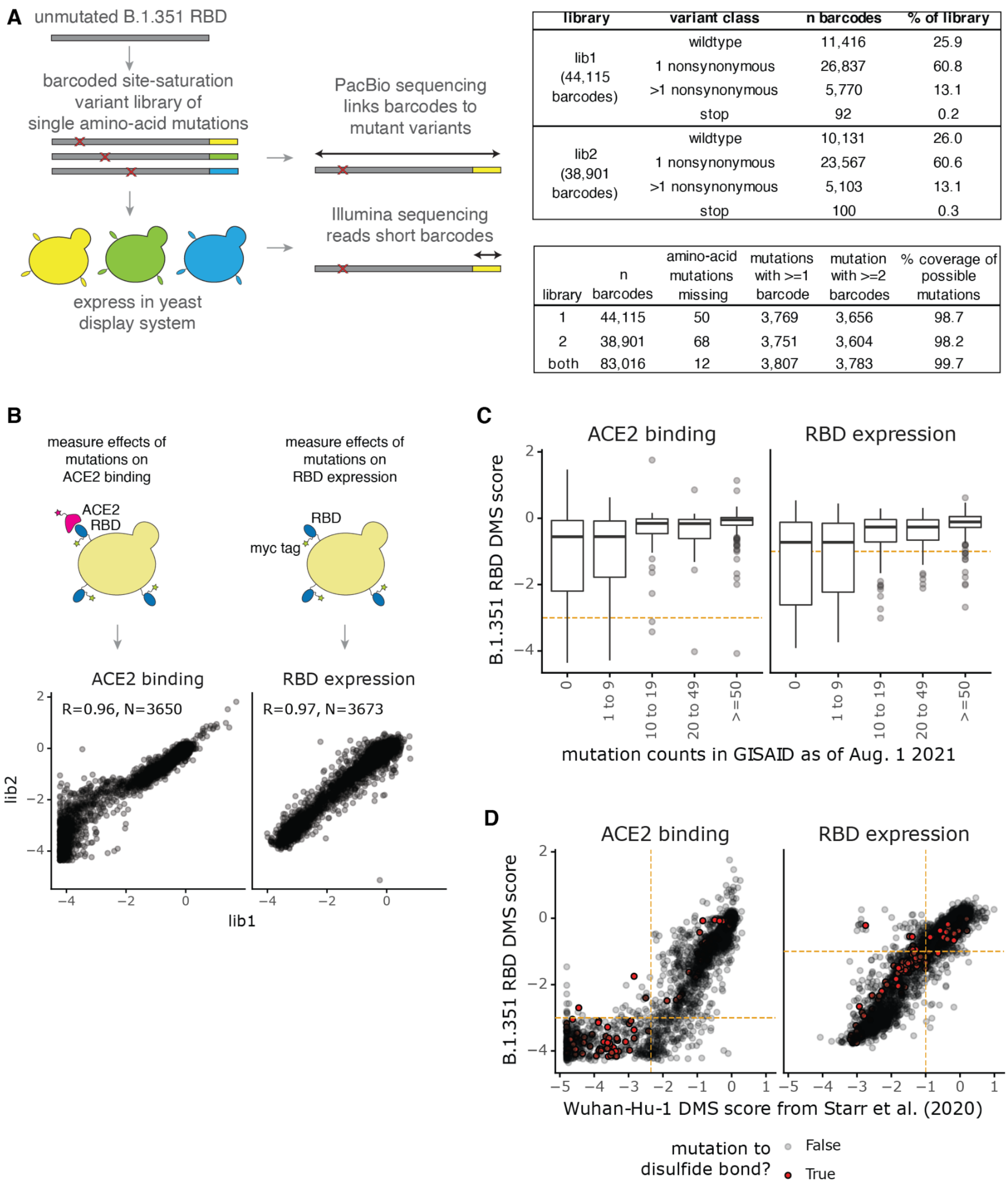

library	variant class	n barcodes	% of library
lib1 (44,115 barcodes)	wildtype	11,416	25.9
	1 nonsynonymous	26,837	60.8
	>1 nonsynonymous	5,770	13.1
	stop	92	0.2
lib2 (38,901 barcodes)	wildtype	10,131	26.0
	1 nonsynonymous	23,567	60.6
	>1 nonsynonymous	5,103	13.1
	stop	100	0.3

library	n barcodes	amino-acid mutations missing	mutations with >=1 barcode	mutation with >=2 barcodes	% coverage of possible mutations
1	44,115	50	3,769	3,656	98.7
2	38,901	68	3,751	3,604	98.2
both	83,016	12	3,807	3,783	99.7

bioRxiv preprint doi: https://doi.org/10.1101/2021.10.12.464114; this version posted October 13, 2021. The copyright holder for this preprint (which was not certified by peer review) is the author/funder, who has granted bioRxiv a license to display the preprint in perpetuity. It is made available under aCC-BY 4.0 International license.

Fig. S2. Generation of the B.1.351 RBD mutant libraries and measurements of effects of mutations on ACE2 binding and RBD expression. (A) Schematic showing the B.1.351 RBD mutant library design. A site-saturation variant library was generated in the B.1.351 RBD background, targeting one amino-acid mutation per variant. Nx16 unique DNA barcodes were added to the variant gene fragments. The Nx16 barcodes were linked to their associated RBD mutations by PacBio circular consensus sequencing (CCS). The plasmid library DNA was transformed into yeast cells. In downstream experiments, the Nx16 barcodes are sequenced by short-read Illumina sequencing. The tables at right indicate key library statistics. **(B)** Correlations between biological independent replicate library measurements of the effects of single mutations on ACE2 binding and RBD expression, measured as described in (*25*). See **Methods** for experimental details. **(C)** Thresholds on the ACE2 binding and RBD expression scores (dashed orange lines) for the B.1.351 mutant library to computationally filter highly deleterious variants that may represent spurious antibody-escape mutations. Importantly, we aimed to retain most mutations that have been observed >=50 times in sequenced SARS-CoV-2 isolates. The x-axis categorizes mutations by their number of observations in GISAID (*81*) as of Aug. 1, 2021. An ACE2 binding score threshold of >= –3.0 (1,000-fold loss in binding affinity) and an RBD expression score of >= –1.0 (10-fold loss in RBD expression) were chosen, which filter comparable numbers of mutations as in prior Wuhan-Hu-1 experiments (*28*, *73*). These filters retain 99.4 and 93.8% of mutations, respectively, that have been observed >=50 times in sequenced SARS-CoV-2 isolates. **(D)** Relationship between the ACE2 binding and RBD expression scores for the B.1.351 RBD library compared to those previously published for the Wuhan-Hu-1 library (*25*). The computational filters used for antibody-escape experiments for the Wuhan-Hu-1 (*28*) and B.1.351 libraries are dashed orange lines. Each dot is one mutation, and mutations to disulfide bonds are shown in red. A key difference is that for the previously published Wuhan-Hu-1 experiments, dimeric rather than monomeric ACE2 was used (*25*).

bioRxiv preprint doi: https://doi.org/10.1101/2021.10.12.464114; this version posted October 13, 2021. The copyright holder for this preprint (which was not certified by peer review) is the author/funder, who has granted bioRxiv a license to display the preprint in perpetuity. It is made available under aCC-BY 4.0 International license.

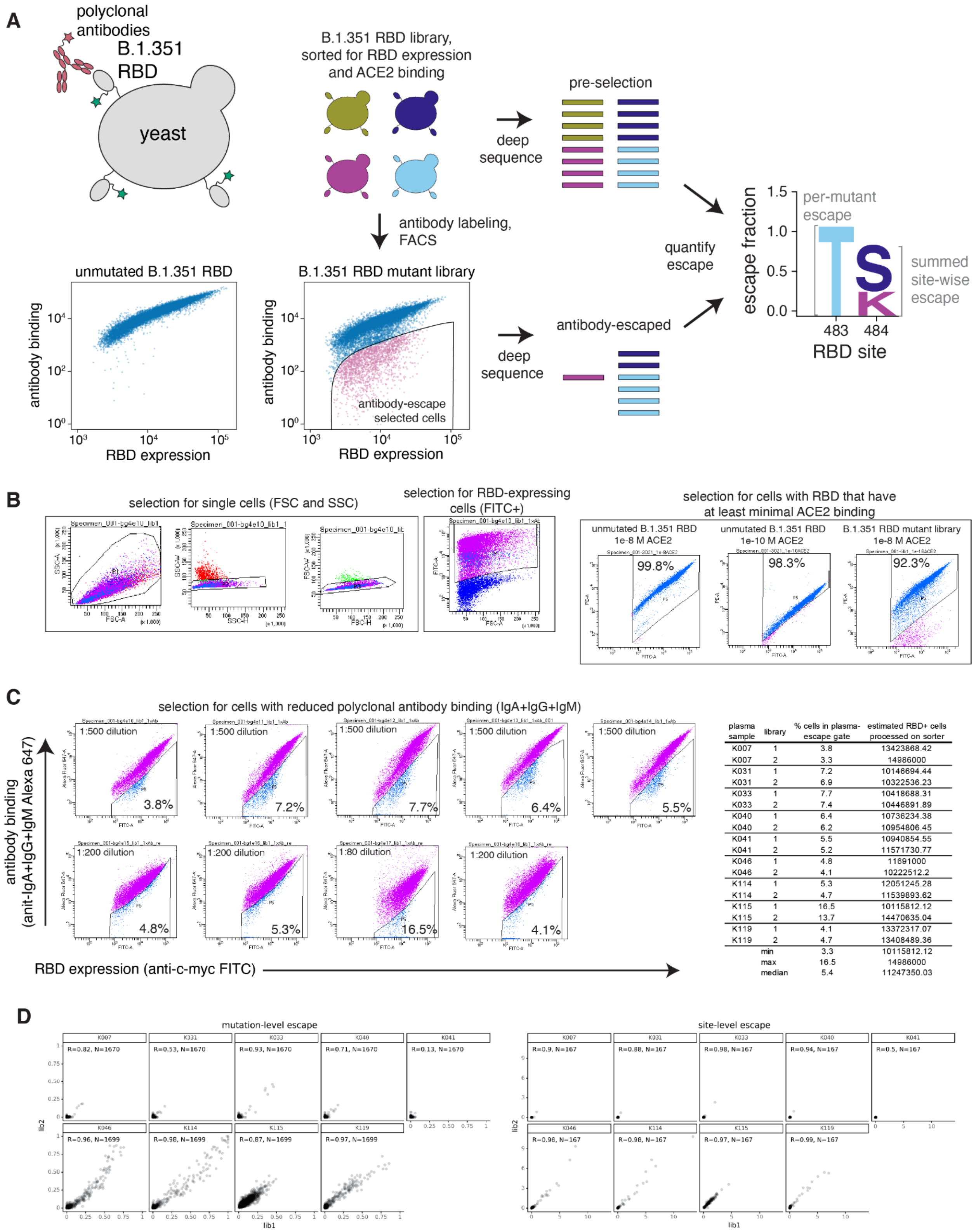

plasma sample	library	% cells in plasma-escape gate	estimated RBD+ cells processed on sorter
K007	1	3.8	13423868.42
K007	2	3.3	14986000
K031	1	7.2	10146694.44
K031	2	6.9	10322536.23
K033	1	7.7	10418688.31
K033	2	7.4	10446891.89
K040	1	6.4	10736234.38
K040	2	6.2	10954806.45
K041	1	5.5	10940854.55
K041	2	5.2	11571730.77
K046	1	4.8	11691000
K046	2	4.1	10222512.2
K114	1	5.3	12051245.28
K114	2	4.7	11539893.62
K115	1	16.5	10115812.12
K115	2	13.7	14470635.04
K119	1	4.1	13372317.07
K119	2	4.7	13408489.36
	min	3.3	10115812.12
	max	16.5	14986000
	median	5.4	11247350.03

bioRxiv preprint doi: https://doi.org/10.1101/2021.10.12.464114; this version posted October 13, 2021. The copyright holder for this preprint (which was not certified by peer review) is the author/funder, who has granted bioRxiv a license to display the preprint in perpetuity. It is made available under aCC-BY 4.0 International license.

Fig. S3. Deep mutational scanning approach to map mutations that reduce binding of B.1.351 infection-elicited polyclonal plasma antibodies to the B.1.351 RBD. (A) Schematic of the approach. The RBD is expressed on the surface of yeast (top left). Flow cytometry is used to quantify both RBD expression (via a C-terminal MYC tag, green star) and antibody binding to the RBD protein expressed on the surface of each yeast cell (bottom left). A library of yeast expressing B.1.351 RBD mutants was incubated with convalescent plasmas and fluorescence-activated cell sorting (FACS) was used to enrich for cells expressing RBD that bound reduced amounts of plasma antibodies, as detected using an IgA+IgG+IgM secondary antibody. Deep sequencing was used to quantify the frequency of each mutation in the initial and antibody-escape cell populations. We quantified the effect of each mutation as the escape fraction, which represents the fraction of cells expressing RBD with that mutation that fell in the antibody escape FACS bin. Escape fractions are represented in logo plots, with the height of each letter proportional to the effect of that amino-acid mutation on antibody binding. The site-level escape metric is the sum of the escape fractions of all mutations at a site. Experimental and computational filtering were used to remove RBD mutants that were misfolded or unable to bind the ACE2 receptor. **(B)** Left: Representative plots of nested FACS gating strategy used for all plasma selection experiments to select for single cells. Samples were gated by SSC-A versus FSC-A, SSC-W versus SSC-H, and FSC-W versus FSC-H) that also express RBD (FITC-A vs. FSC-A). Right: The RBD mutant libraries were sorted to retain cells expressing variants that bound to ACE2 with at least nominal affinity. Unmutated B.1.351 RBD and each RBD mutant library was incubated with dimeric ACE2 at 10^{-8} M. A FACS selection gate was set to capture 98% of cells expressing unmutated B.1.351 RBD that were incubated with 10^{-10} M ACE2, to purge the mutant libraries of highly deleterious mutations (i.e., those that have <1% the affinity of unmutated B.1.351 RBD). **(C)** Left: FACS gating strategy for one of two independent libraries to select cells expressing RBD mutants with reduced binding by polyclonal sera (cells in blue). Gates were set manually during sorting. Selection gates were set to capture ~5% of the RBD+ library. The same gate was set for both independent libraries stained with each plasma sample, and the FACS scatter plots looked qualitatively similar between the two libraries. Right: the fraction of library cells that fall into each selection gate. **(D)** Mutation- and site-level correlations of escape scores between biologically independent library replicates. SSC-A, side scatter-area; FSC-A, forward scatter-area; SSC-W, side scatter-width; SSC-H, side scatter-height; FSC-W, forward scatter-width; FSC-H, forward scatter height; FITC-A, fluorescein isothiocyanate-area.

bioRxiv preprint doi: https://doi.org/10.1101/2021.10.12.464114; this version posted October 13, 2021. The copyright holder for this preprint (which was not certified by peer review) is the author/funder, who has granted bioRxiv a license to display the preprint in perpetuity. It is made available under aCC-BY 4.0 International license.

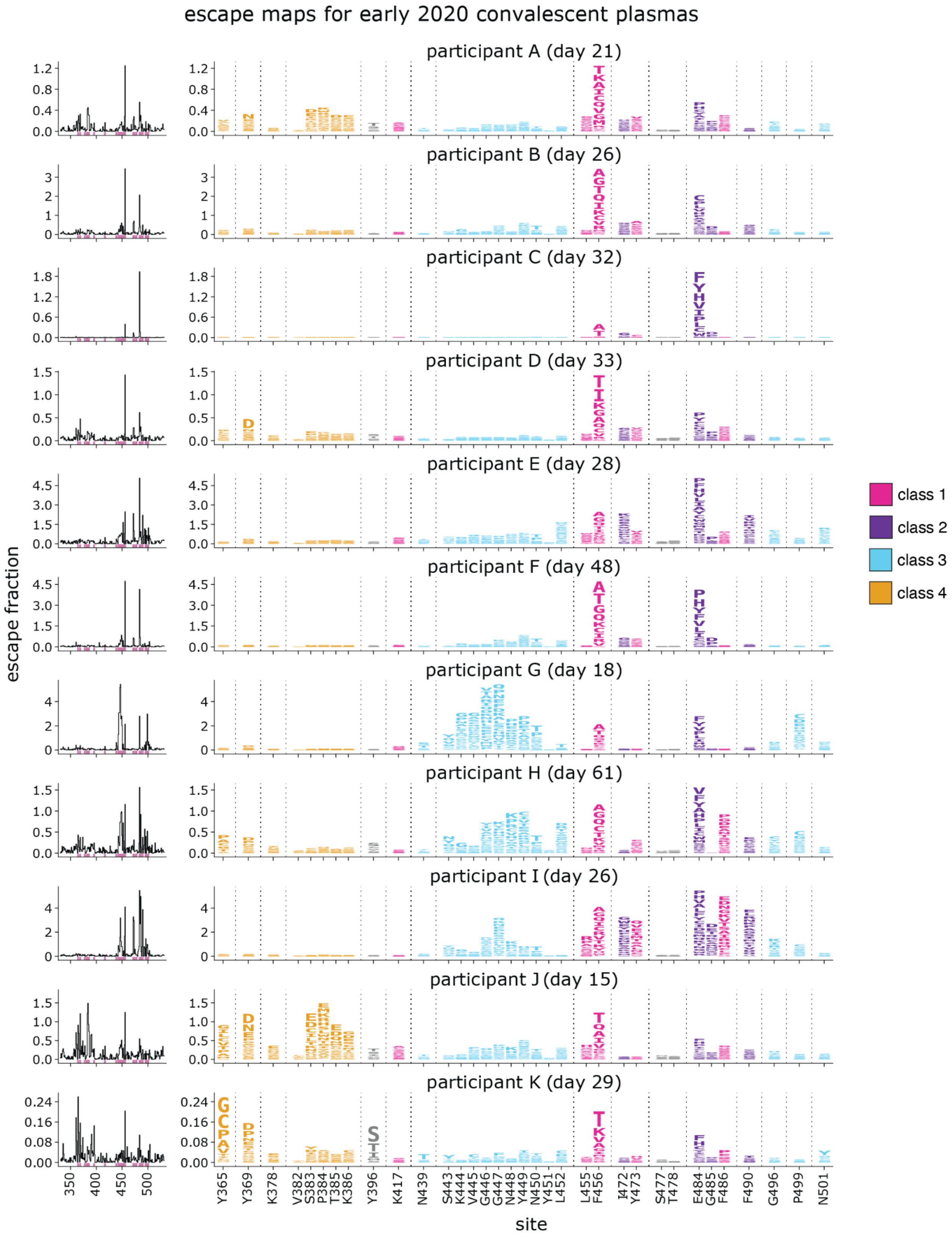

bioRxiv preprint doi: https://doi.org/10.1101/2021.10.12.464114; this version posted October 13, 2021. The copyright holder for this preprint (which was not certified by peer review) is the author/funder, who has granted bioRxiv a license to display the preprint in perpetuity. It is made available under aCC-BY 4.0 International license.

Fig. S4. Escape maps for the early 2020 convalescent plasmas, as measured using a deep mutational scanning approach in the Wuhan-Hu-1 RBD background. The line plots at left indicate the site-level antibody escape for all RBD sites, and the logo plots at right zoom in on key sites (highlighted in purple on the line plot x-axes). For each sample, the y-axis is scaled independently. RBD sites are colored by antibody epitope. Sites 417, 484, and 501 are labeled with red text on the x-axis. All 11 samples from the Washington state early 2020 cohort (*28*) are shown here and averaged in **Fig. 4**. Interactive versions of logo plots and structural visualizations are at https://jbloomlab.github.io/SARS-CoV-2-RBD_B.1.351/. These data were originally published in (*28*) and are reanalyzed here. The numerical antibody-escape scores are in **data file S4** and at https://github.com/jbloomlab/SARS-CoV-2-RBD_B.1.351/blob/main/results/prior_DMS_data/early2020_escape_fracs.csv.

bioRxiv preprint doi: https://doi.org/10.1101/2021.10.12.464114; this version posted October 13, 2021. The copyright holder for this preprint (which was not certified by peer review) is the author/funder, who has granted bioRxiv a license to display the preprint in perpetuity. It is made available under aCC-BY 4.0 International license.

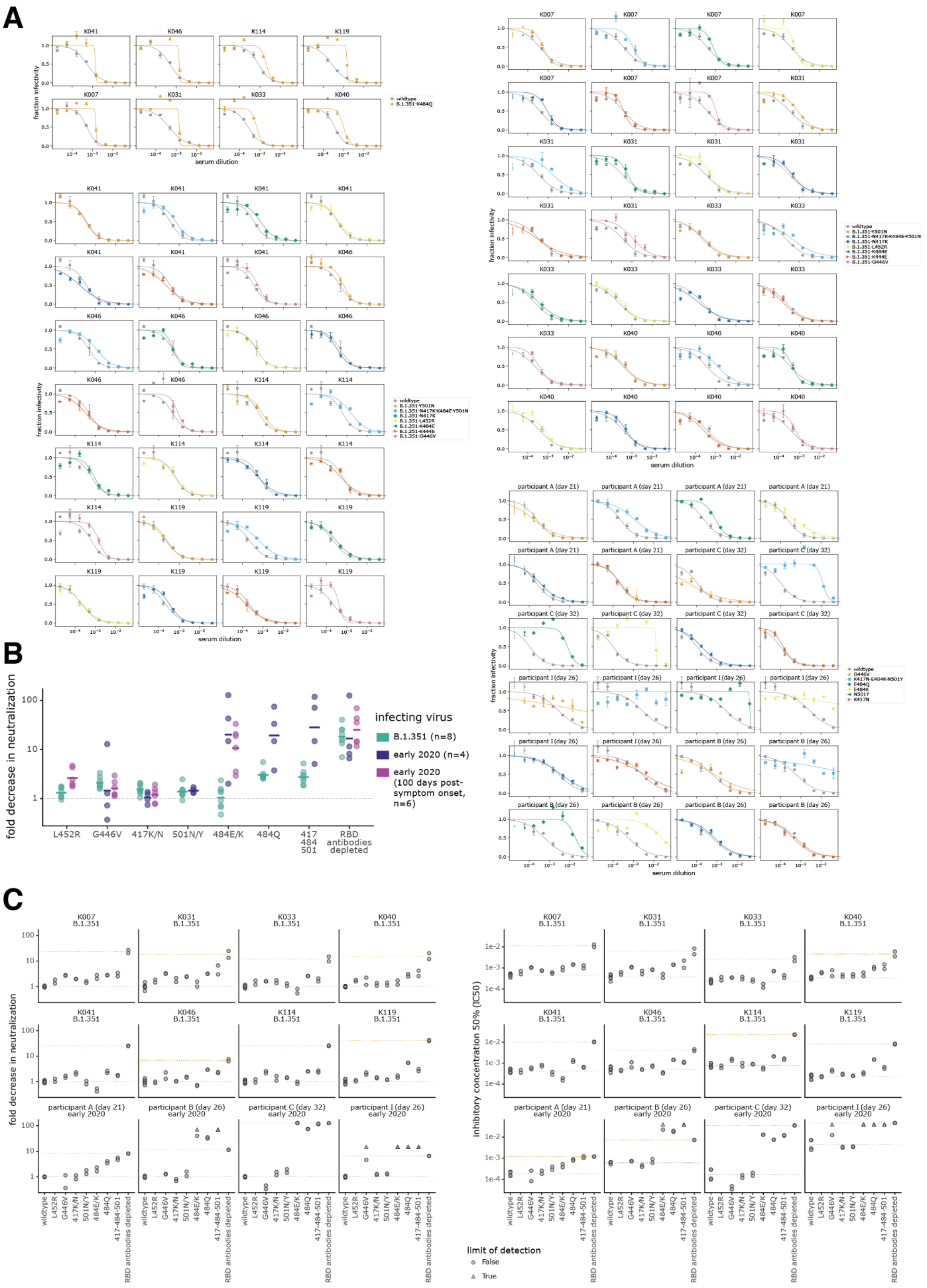

bioRxiv preprint doi: https://doi.org/10.1101/2021.10.12.464114; this version posted October 13, 2021. The copyright holder for this preprint (which was not certified by peer review) is the author/funder, who has granted bioRxiv a license to display the preprint in perpetuity. It is made available under aCC-BY 4.0 International license.

Fig. S5. Neutralization of point mutants of B.1.351 and D614G spike-pseudotyped lentiviral particles by convalescent plasmas from B.1.351 and early 2020-infected individuals. (A) Each plot shows the neutralization curves of one point mutant and the wildtype measured on the same assay date for each plasma (the same wildtype curve is repeated on multiple plots for comparison). Plots are grouped by assay date. Each point is the average of two technical replicates. **(B)** The fold-decrease in neutralization for samples shown in **Fig. 5**, with the addition of previously measured neutralization by samples from 6 early 2020 convalescent individuals collected approximately 100 days post-symptom onset (*38*). **(C)** The fold-change in IC50 (left) or the absolute IC50 (right) for the neutralization of each point mutant by each plasma. The fold-change IC50 is calculated relative to the geometric mean of two wildtype technical replicates performed on the same assay date. Each point is one technical replicate. The dashed gray line indicates the geometric mean of all wildtype measurements for that plasma, and the orange line indicates the geometric mean of the effect of removing all RBD-binding antibodies. B.1.351 plasma names are prefixed with K*, and early 2020 plasmas are prefixed with "participant". All assays were performed with the "homologous" virus: B.1.351 spike for B.1.351 plasmas, and D614G spike for early 2020 plasmas. Mutations are given the same names for B.1.351 and D614G spikes, so 417K/N is 417N in the B.1.351 background and 417K in the D614G background; 484E/K is 484E in B.1.351 and 484K in D614G; 501N/Y is 501N in B.1.351 and 501Y in D614G; and 417-484-501 is 417K-484E-501N in B.1.351 and 417N-484K-501Y in D614G.
